# Association Between DMFT Index, Dental Plaque, and Pre‐eclampsia in Pregnant Women: A Case–Control Study

**DOI:** 10.1002/cre2.70177

**Published:** 2025-07-17

**Authors:** Mehdi Shirinzad, Mohammad Moradi Dalir, Azita Tiznobaik, Farideh Kazemi, Zohreh Momenimovahed

**Affiliations:** ^1^ Department of Restorative Dentistry, School of Dentistry Hamadan University of Medical Sciences Hamadan Iran; ^2^ Mother and Child Care Research Center, Institute of Health Sciences and Technologies Hamadan University of Medical Sciences Hamadan Iran; ^3^ Department of Midwifery and Reproductive Health, School of Nursing and Midwifery Hamadan University of Medical Sciences Hamadan Iran; ^4^ Department of Midwifery, School of Medicine Qom University of Medical Sciences Qom Iran

**Keywords:** dental plaque, DMFT index, pre‐eclampsia, pregnancy

## Abstract

**Objectives:**

This study investigates the association between Decayed, Missing, and Filled Teeth (DMFT) Index, dental plaque (assessed via Plaque Index [PI]), and pre‐eclampsia in pregnant women.

**Material and Methods:**

A case–control study was conducted with 70 pregnant women diagnosed with pre‐eclampsia (cases) and 70 without (controls), matched for demographic and obstetric variables. Data were collected using a checklist capturing demographic details, obstetric history, and oral health parameters (DMFT and PI). Statistical analyses included chi‐square tests, independent *t*‐tests, and multiple logistic regression.

**Results:**

The case group exhibited a significantly higher mean DMFT index (11.70 ± 2.88 vs. 9.21 ± 2.24, *p* < 0.001) and PI (33.26 ± 9.21 vs. 28.30 ± 7.26, *p* < 0.001) compared to controls. Each one‐unit increase in the DMFT index and PI was associated with 19% and 5% increased odds of pre‐eclampsia, respectively (*p* < 0.001).

**Conclusions:**

Dental caries and plaque are significantly associated with pre‐eclampsia in pregnant women. Integrating oral health assessments into antenatal care is recommended to mitigate these risks and improve maternal and fetal outcomes.

## 1 Introduction

Pre‐eclampsia, a multisystem hypertensive disorder affecting approximately 4.6% of pregnancies globally, is a major contributor to maternal and fetal morbidity and mortality. In Iran, recent estimates indicate a prevalence of 5%, reflecting an increasing trend (Abdollahpour et al. 2024). Characterized by hypertension (blood pressure ≥ 140/90 mmHg) and proteinuria after 20 weeks of gestation, pre‐eclampsia is associated with severe complications, including heart failure, pulmonary edema, and hemolysis, elevated liver enzymes, and low platelet (HELLP) syndrome (Phipps et al. 2016). Although its etiology remains unclear, potential contributors include inadequate uterine blood flow, vascular damage, and immune dysregulation (Wheeler et al. 2022). Mild and severe are the two types of pre‐eclampsia. The severe type is characterized by diastolic blood pressure of 110 mmHg or more, double positive proteinuria or more, high creatinine, elevated liver enzymes and headache, allergy, pulmonary edema, upper abdominal pain, visual disturbances, and thrombocytopenia (Nirupama et al. 2021). One of the serious complications of pre‐eclampsia is end‐organ involvement in the form of heart failure, pulmonary edema, liver and coagulation system involvement, and finally HELLP syndrome, brain involvement, convulsions, and even maternal death. On the other hand, due to problems in placental‐uterine bleeding, there is a possibility of intrauterine growth retardation (IUGR) and intrauterine fetal death (IUFD) (Phipps et al. 2016).

Periodontal disease, characterized by microbial plaque‐induced inflammation, has a significant burden in Iran, with studies reporting that 30%–53% of adults (Ayoobi et al. 2023). Many epidemiological studies have found a positive association between periodontal disease and pre‐eclampsia (Soucy‐Giguère et al. 2016). Studies have indicated that pregnancy can increase the risk of tooth decay because of changes in the pH and buffering capacity of saliva, as well as unhealthy eating habits and failure to observe complete oral hygiene, including the use of toothbrushes and dental floss, due to the emotional changes and habits of pregnant women during this period, as well as hormonal changes in pregnant women, such as increased cortisol, which prepares the oral environment for the activity of cariogenic bacteria and increased dental plaque (Tiznobaik et al. 2019). Dental caries, sharing a common etiology with periodontal disease, affects 50–87% of pregnant women globally (Caracho et al. 2020). During pregnancy, the inflammatory response is characterized by the expression of various inflammatory mediators. During pregnancy, complications can arise due to the intensification of this inflammatory response. The risk of pre‐eclampsia, prematurity, and low birth weight babies may be increased by subgingival bacteria from the oral cavity entering the mother's bloodstream (Daalderop et al. 2018). On the other hand, interestingly, both periodontal diseases and dental caries have the same etiology, which is microbial plaque (Jaiman et al. 2018). In dentistry, it has been proven that any infection in the oral environment, including microbial plaques, can cause harm to distant parts of the body (Rahmani et al. 2013). Given the common origin of periodontal disease and dental caries, and the fact that any infection in the oral area can have adverse effects in distant parts of the body, the authors of this article believe that dental caries can also cause pre‐eclampsia in pregnant mothers, as previously observed in periodontal diseases. Few studies have been conducted on the relationship between dental caries and pre‐eclampsia. Given the prevalence of caries in all human societies, especially its very high prevalence in developing societies, and the importance of women's health, especially during pregnancy and childbirth, as an important indicator of human development, Therefore, the present study was conducted with the aim of determining the relationship between DMFT indices and dental plaque with pre‐eclampsia in pregnant women referring to comprehensive health centers in Hamadan.

## 2 Materials and Methods

### 2.1 Study Design and Population

This case–control study was conducted in 2022 among pregnant women attending maternal health clinics at comprehensive health centers in Hamadan, Iran. Participants were selected using cluster sampling from a population of eligible pregnant women meeting predefined inclusion criteria.

### 2.2 Sample Size and Sampling

Sample size was calculated using the Sampsi module in Stata‐13, based on Rahmani et al.'s (2013) study with 90% power, 5% error, and proportions of p1 = 0 and p2 = 0.167, yielding 70 participants per group (cases and controls). Hamadan was divided into four regions, and three health centers were randomly selected from each region. Cases (pregnant women with pre‐eclampsia) and controls (without pre‐eclampsia) were equally recruited from these 12 centers based on inclusion and exclusion criteria.

### 2.3 Inclusion and Exclusion Criteria

Eligible participants were pregnant women aged < 35 years, with a gestational age of 20–34 weeks, and a minimum of 25 teeth. Cases were diagnosed with pre‐eclampsia by an obstetrician, based on blood pressure ≥ 140/90 mmHg and proteinuria ( > 300 mg/24‐h urine or ≥ 30 mg/dL in a random sample). Exclusion criteria included diabetes, chronic hypertension, chronic liver or kidney disease, severe dental caries requiring antibiotic therapy, obesity (BMI ≥ 30), recent dental treatment (within 6 months before pregnancy), oral habits, or conditions requiring antibiotic prophylaxis.

### 2.4 Study Implementation

Seventy women with pre‐eclampsia (cases) and 70 without (controls) were enrolled, matched for demographic and obstetric variables (e.g., age, gestational age, marital duration). A dental student, under supervision, conducted oral examinations using World Health Organization (WHO) criteria (WHO 2013). Dental caries was assessed visually and with a probe, identifying lesions in pits, fissures, or smooth surfaces with softened enamel. Plaque Index (PI) was calculated as a percentage using GUM Red‐Cote Disclosing Plaque Tablets (Mclaren, Australia), by dividing the number of surfaces with visible biofilm by the total surfaces examined and multiplying by 100.

### 2.5 Data Collection

Data were collected using a three‐part checklist: (1) demographic information (age, weight, height, education, occupation, spouse's occupation), (2) obstetric history (gestational age, parity, abortion history), and (3) oral health parameters (DMFT index and PI), based on the visible dental plaque index, adhering to WHO guidelines (WHO 2013).

### 2.6 Statistical Analysis

Data were analyzed using Stata‐13. Descriptive statistics included means and standard deviations for continuous variables and frequencies and percentages for categorical variables. The Kolmogorov‐Smirnov test assessed data normality. Group comparisons used independent *t*‐tests for continuous variables and chi‐square tests for categorical variables. Multiple logistic regression evaluated the association between DMFT, PI, and pre‐eclampsia, adjusting for confounders (age, gestational age, marital duration). A significance level of *p* < 0.05 was applied.

### 2.7 Ethical Considerations

The study was approved by the Ethics Committee of Hamadan University of Medical Sciences (IR.UMSHA.REC.1401.197). Participants provided written informed consent, and confidentiality was ensured.

## 3 Results

### 3.1 Demographic Characteristics

No significant differences were observed between cases and controls in demographic variables, including age (27.12 ± 2.6 vs. 28.42 ± 3.1 years, *p* = 0.20), marital age, occupation, spouse's occupation, education, or household income (*p* > 0.05; Table 1), confirming effective matching.

**Table 1:** Demographic characteristics by study group.

Variable	Case (*N* = 70)	Control (*N* = 70)	*p* value*
**Age (years, mean ± SD)**	27.12 ± 2.6	28.42 ± 3.1	0.20
**Marriage age (years, mean ± SD)**	22.4 ± 2.1	21.87 ± 1.9	0.51
**Occupation, *n* (%)**			0.14
Housewife	51 (72.8)	63 (90)	
Employed	19 (27.2)	7 (0)	
**Spouse's occupation, *n* (%)**			0.26
Unemployed	5 (7.2)	0 (0.0)	
Employed	65 (92.8)	70 (100)	
**Education, *n* (%)**			0.43
Illiterate	10 (14.2)	7 (10)	
Less than high school	25 (35.2)	27 (38.6)	
High school	27 (38.6)	30 (42.3)	
University	8 (11.2)	6 (8.6)	
**Spouse's education, *n* (%)**			0.60
Illiterate	5 (7.1)	2 (2.8)	
Less than high school	18 (25.7)	23 (32.8)	
High school	37 (52.9)	41 (58.6)	
University	10 (14.3)	4 (5.7)	
**Household income, *n* (%)**			0.74
≥ $85 per month	11 (15.7)	6 (8.6)	
< $85 per month	59 (84.3)	64 (91.4)	

* Chi‐square test for categorical variables, independent *t*‐test for continuous variables.

### 3.2 Dental and Oral Hygiene Variables

No significant differences were found in dental visit frequency, oral hygiene practices before pregnancy, or oral hygiene during pregnancy between groups (*p* > 0.05; Table 2), indicating comparable oral hygiene behaviors.

**Table 2:** Dental and oral hygiene variables by study group.

Variable	Case (*N* = 70), *n* (%)	Control (*N* = 70), *n* (%)	*p* value*
**Dental visit frequency**			0.248
Every 3 months	2 (2.8)	4 (1.4)	
Every 6 months	8 (11.4)	3 (4.3)	
Once a year	12 (17.2)	11 (5.7)	
Only when in pain	48 (68.6)	55 (87.6)	
**Oral hygiene before pregnancy**			0.118
Toothbrush once/day	35 (50.0)	36 (51.4)	
Toothbrush twice/day	16 (22.9)	13 (18.6)	
Toothbrush three times/day	8 (4.4)	6 (8.6)	
Toothbrush + floss	6 (14.3)	8 (11.4)	
Toothbrush + mouthwash	5 (7.1)	6 (8.6)	
Toothbrush + floss + mouthwash	1 (1.4)	1 (1.4)	
**Oral hygiene during pregnancy**			0.188
Toothbrush once/day	22 (31.4)	28 (40.0)	
Toothbrush twice/day	23 (32.9)	20 (28.6)	
Toothbrush three times/day	4 (5.7)	2 (2.9)	
Toothbrush + floss	8 (11.4)	7 (10.0)	
Toothbrush + mouthwash	2 (2.9)	2 (2.9)	
Toothbrush + floss + mouthwash	1 (1.4)	1 (1.4)	
None	10 (14.3)	10 (14.3)	

* Chi‐squared test.

### 3.3 Gynecological and Obstetric Characteristics

No significant difference was found in primiparity between groups (cases: 74.3%, controls: 48.6%, *p* = 0.06). As expected, all case group participants (100%) had blood pressure ≥ 140/90 mmHg and proteinuria at ≥ 30 weeks' gestation, compared to none (0%) in the control group (40%), confirming the pre‐eclampsia diagnosis in cases.

### 3.4 Dental Caries and Plaque Variables

The total number of teeth was similar between groups (28.65 ± 2.40 vs. 28.53 ± 2.11, *p* = 0.47). Significant differences were observed in healthy teeth (18.87 ± 2.91 vs. 20.71 ± 3.45, *p* < 0.001), decayed teeth (5.67 ± 2.53 vs. 3.17 ± 2.16, *p* < 0.001), DMFT index (11.70 ± 2.88 vs. 9.21 ± 2.24, *p* < 0.001), and PI (33.26 ± 9.21 vs. 28.30 ± 7.26, *p* < 0.001). No differences were found in missing (*p* = 0.67) or filled teeth (*p* = 0.16; Table 3).

**Table 3:** Dental caries and plaque variables by study group.

Variable (mean ± SD)	Case (*N* = 70)	Control (*N* = 70)	*p* value*
Total teeth	28.65 ± 2.40	28.53 ± 2.11	0.47
Healthy teeth	18.87 ± 2.91	20.71 ± 3.45	< 0.001
Decayed (D)	5.67 ± 2.53	3.17 ± 2.16	< 0.001
Missing (M)	2.60 ± 2.04	2.53 ± 2.26	0.67
Filled (F)	3.84 ± 3.44	3.72 ± 3.13	0.16
DMFT Index	11.70 ± 2.88	9.21 ± 2.24	< 0.001
Plaque Index (PI)	33.26 ± 9.21	28.30 ± 7.26	< 0.001

* Independent *t*‐test.

Multiple logistic regression revealed that each one‐unit increase in decayed teeth, DMFT index, and PI was associated with a 51% (OR 1.51, 95% CI 1.23–1.82), 19% (OR 1.19, 95% CI 1.03–1.31), and 5% (OR 1.05, 95% CI 1.02–1.09) increased odds of pre‐eclampsia, respectively (*p* < 0.001; Table 4).

**Table 4:** Probability ratio of pre‐eclampsia due to DMFT and Plaque Index.

Variable	Adjusted OR (95% CI)	*p* value*
Decayed (D)	1.51 (1.23–1.82)	< 0.001
Missing (M)	0.92 (0.77–1.13)	0.67
Filled (F)	1.07 (0.96–1.29)	0.16
DMFT Index	1.19 (1.03–1.31)	< 0.001
Plaque Index (PI)	1.05 (1.02–1.09)	< 0.001

* Multiple logistic regression, adjusted for age, gestational age, and marital duration.

## 4 Discussion

This study identified a significant association between dental caries, microbial plaque, and pre‐eclampsia in pregnant women. The case group exhibited a higher mean number of decayed teeth (5.67 ± 2.53 vs. 3.17 ± 2.16, *p* < 0.001), DMFT index (11.70 ± 2.88 vs. 9.21 ± 2.24, *p* < 0.001), and PI (33.26 ± 9.21 vs. 28.30 ± 7.26, *p* < 0.001) compared to controls. Multiple logistic regression confirmed that each one‐unit increase in decayed teeth, DMFT index, and PI was associated with a 51% (OR 1.51, 95% CI 1.23–1.82), 19% (OR 1.19, 95% CI 1.03–1.31), and 5% (OR 1.05, 95% CI 1.02–1.09) increased odds of pre‐eclampsia, respectively (*p* < 0.001).

In dentistry, two major diseases exist: periodontal diseases and dental caries, both of which are among the most common oral health issues. Interestingly, both share a common origin, namely microbial plaque. In recent years, some dental researchers have investigated the relationship between periodontal diseases and adverse pregnancy outcomes, and recently, attention has been drawn to the association between periodontal diseases and pre‐eclampsia. Furthermore, in dentistry, it has been established that any infection in the oral environment, as well as microbial plaque, can have undesirable effects on distant parts of the body (Soucy‐Giguère et al. 2016).

In the study by Barak and colleagues, a correlation between pre‐eclampsia and periodontitis was confirmed. In this study, in addition to dental plaque, pocket depth and clinical attachment loss (CAL) were also measured. However, dental caries, which is directly related to dental plaque, was not considered (Barak et al. 2007).

In the study by Farhoodi and colleagues, a strongly significant relationship was observed between microbial plaque and pre‐eclampsia, which is consistent with the results of our study (Farhoodi et al. 2021).

In the study by Horton and colleagues, no correlation was found between periodontal disease and pre‐eclampsia. The difference in findings between Horton et al. and the present study likely stems from methodological variations, particularly in the measurement of periodontal plaque. Horton et al.'s qualitative assessment (categorizing periodontal status into healthy, mild, moderate, and severe) may lack the granularity needed to detect subtle associations between microbial plaque and pre‐eclampsia. Qualitative classifications can introduce subjectivity and reduce statistical power, especially if the categories do not capture the full spectrum of plaque‐related pathology. In contrast, the present study's quantitative approach counting teeth affected by microbial plaque—provides a more precise and objective metric, likely increasing sensitivity to detect correlations. This methodological rigor aligns with the significant association found between microbial plaque and pre‐eclampsia in the present study and Farhoudi et al.'s findings (Horton et al. 2010).

In the study by Abati and colleagues, no association was found between DMFT (Decayed, Missing, and Filled Teeth) index and preterm birth, low birth weight, or pre‐eclampsia. The lack of association reported by Abati et al. may be attributed to the limited sample size of the case group (*n* = 62), which likely reduced the statistical power to detect significant correlations between DMFT and adverse pregnancy outcomes (preterm birth, low birth weight, and pre‐eclampsia). In clinical research, small sample sizes can lead to type II errors (failing to detect a true effect), particularly when studying multifactorial conditions like pre‐eclampsia, where effect sizes may be modest. The imbalance between the control group (*n* = 242) and the case group (*n* = 62) further complicates the analysis, as it may skew statistical comparisons and limit the generalizability of findings (Abati et al. 2013).

The study's robustness is enhanced by its sufficient sample size, determined through statistical power calculations, which ensures adequate statistical power to detect significant differences between the case and control groups. Additionally, the matching of participants across demographic, obstetric, and dental variables (with *p* > 0.05, indicating no significant differences in these characteristics) minimizes confounding factors, increasing the validity of the observed associations (e.g., between microbial plaque and pre‐eclampsia). This methodological rigor strengthens the reliability and generalizability of the findings.

From a pathophysiological perspective, it has been suggested that cariogenic pathogens may contribute through a direct inflammatory effect or via indirect mediator effects induced by the microbes (Larvin et al. 2021). The inflammatory response is naturally activated during pregnancy, characterized by the expression of various inflammatory mediators. Exacerbation of this inflammatory response can lead to pregnancy complications. It has also been hypothesized that pathogenic bacteria present in the oral cavity may enter the bloodstream (Soucy‐Giguère et al. 2016).

Pre‐eclampsia is characterized by multiple unclear factors, including genetic and environmental components, and is associated with an underlying inflammatory disorder. The inflammatory response related to pregnancy and pre‐eclampsia can be assessed by measuring the cytokine profile in the bloodstream (Cakir et al. 2020).

Several studies support the possibility of oral bacteria or their bacterial products spreading through the bloodstream to the placenta. In a microbiological assessment, the total count of various anaerobic bacteria in blood and placental samples was higher in the pre‐eclampsia group compared to the group without pre‐eclampsia. Thus, multiple pathogens may contribute to the risk of pre‐eclampsia rather than a single pathogen (Mahendra et al. 2021). Additionally, a higher concentration of TNF‐α in the serum of patients with pre‐eclampsia compared to those without pre‐eclampsia was found, with statistically significant differences using both ELISA and PCR methods (Aggarwal et al. 2019). The increased TNF‐α level simply reflects the natural progression of the inflammatory cascade.

## 5 Strengths and Limitations

This study's strengths include its adequate sample size, calculated for 90% power, and rigorous matching of cases and controls, which minimized confounding factors. Potential biases, such as recall bias in reporting oral hygiene practices, were reduced through standardized data collection; however, residual confounding cannot be fully excluded. Limitations include the lack of assessment of multifactorial contributors to pre‐eclampsia (e.g., genetic or environmental factors) and reliance on clinical rather than biochemical markers of inflammation. Generalizability may be limited to populations with similar demographic and clinical characteristics. Future studies should replicate these findings and measure blood‐based biomarkers (e.g., cytokines or bacterial products) to elucidate the mechanistic link between oral health and pre‐eclampsia.

## 6 Conclusion

This study demonstrates a significant association between dental caries, microbial plaque, and pre‐eclampsia in pregnant women. Higher DMFT and plaque indices were associated with increased odds of pre‐eclampsia, highlighting the importance of oral health in maternal well‐being. Health policymakers should prioritize integrating oral health assessments and interventions into antenatal care to reduce pre‐eclampsia risk and improve maternal and fetal outcomes.

## Author Contributions

Mehdi Shirinzad and Mohammad Moradi Dalir contributed to the study design and data collection. Azita Tiznobaik supervised the study and wrote the manuscript. Farideh Kazemi and Zohreh Momenimovahed performed the statistical analysis and contributed to manuscript editing. All authors read and approved the final manuscript.

## Conflicts of Interest

The authors declare no conflicts of interest.

## Data Availability

The data that support the findings of this study are available on request from the corresponding author. The data are not publicly available due to privacy or ethical restrictions.

## References

[cre270177-bib-0001] Abati, S. , A. Villa , I. Cetin , et al. 2013. “Lack of Association Between Maternal Periodontal Status and Adverse Pregnancy Outcomes: A Multicentric Epidemiologic Study.” Journal of Maternal‐Fetal & Neonatal Medicine 26, no. 4: 369–372. 23039761 10.3109/14767058.2012.733776

[cre270177-bib-0002] Abdollahpour, S. , T. Khadivzadeh , M. Shafeei , and M. Arian . 2024. “Prevalence of Preeclampsia and Eclampsia in Iran: An Updated Systematic Review and Meta‐Analysis.” Iranian Journal of Nursing and Midwifery Research 29, no. 5: 495–502. 39478718 10.4103/ijnmr.ijnmr_299_23 PMC11521136

[cre270177-bib-0003] Aggarwal, R. , A. K. Jain , P. Mittal , M. Kohli , P. Jawanjal , and G. Rath . 2019. “Association of Pro‐ and Anti‐inflammatory Cytokines in Preeclampsia.” Journal of Clinical Laboratory Analysis 33, no. 4: e22834. 30666720 10.1002/jcla.22834 PMC6528584

[cre270177-bib-0004] Ayoobi, F. , S. Salari Sedigh , P. Khalili , et al. 2023. “Dyslipidemia, Diabetes and Periodontal Disease, a Cross‐Sectional Study in Rafsanjan, a Region in Southeast Iran.” BMC Oral Health 23, no. 1: 549. 37563720 10.1186/s12903-023-03262-x PMC10416538

[cre270177-bib-0005] Barak, S. , O. Oettinger‐Barak , E. E. Machtei , H. Sprecher , and G. Ohel . 2007. “Evidence of Periopathogenic Microorganisms in Placentas of Women With Preeclampsia.” Journal of Periodontology 78, no. 4: 670–676. 17397314 10.1902/jop.2007.060362

[cre270177-bib-0006] Cakir, S. C. , B. A. Dorum , N. Koksal , and H. Ozkan . 2020. “The Effects of Maternal Preeclampsia on Inflammatory Cytokines and Clinical Outcomes in Premature Infants.” Pakistan Journal of Medical Sciences 36, no. 2: 26–31. 32063926 10.12669/pjms.36.2.1316 PMC6994880

[cre270177-bib-0007] Caracho, R. A. , G. A. Foratori‐Junior , N. S. Fusco , B. G. Jesuino , A. L. T. Missio , and S. H. C. Sales‐Peres . 2020. “Systemic Conditions and Oral Health‐Related Quality of Life of Pregnant Women of Normal Weight and Who Are Overweight.” International Dental Journal 70, no. 4: 287–295. 32107768 10.1111/idj.12547 PMC9379178

[cre270177-bib-0008] Daalderop, L. A. , B. V. Wieland , K. Tomsin , et al. 2018. “Periodontal Disease and Pregnancy Outcomes: Overview of Systematic Reviews.” JDR Clinical & Translational Research 3, no. 1: 10–27. 30370334 10.1177/2380084417731097 PMC6191679

[cre270177-bib-0009] Farhoodi, I. , Z. Mortazavi , R. Dargahi , and A. Naghizadeh Baghi . 2021. “Comparing Periodontal Status Between Pregnant Women With Preeclampsia and Normal Women.” Avicenna Journal of Dental Research 13, no. 1: 13–17.

[cre270177-bib-0010] Horton, A. L. , K. A. Boggess , K. L. Moss , J. Beck , and S. Offenbacher . 2010. “Periodontal Disease, Oxidative Stress, and Risk for Preeclampsia.” Journal of Periodontology 81, no. 2: 199–204. 20151797 10.1902/jop.2009.090437 PMC4380012

[cre270177-bib-0011] Jaiman, G. , P. Nayak , S. Sharma , and K. Nagpal . 2018. “Maternal Periodontal Disease and Preeclampsia in Jaipur Population.” Journal of Indian Society of Periodontology 22, no. 1: 50–54. 29568173 10.4103/jisp.jisp_363_15 PMC5855271

[cre270177-bib-0012] Larvin, H. , J. Kang , V. R. Aggarwal , S. Pavitt , and J. Wu . 2021. “Risk of Incident Cardiovascular Disease in People With Periodontal Disease: A Systematic Review and Meta‐Analysis.” Clinical and Experimental Dental Research 7, no. 1: 109–122. 33124761 10.1002/cre2.336 PMC7853902

[cre270177-bib-0013] Mahendra, J. , L. Mahendra , M. H. Mugri , et al. 2021. “Role of Periodontal Bacteria, Viruses, and Placental mir155 in Chronic Periodontitis and Preeclampsia—A Genetic Microbiological Study.” Current Issues in Molecular Biology 43, no. 2: 831–844. 34449559 10.3390/cimb43020060 PMC8929077

[cre270177-bib-0014] Nirupama, R. , S. Divyashree , P. Janhavi , S. P. Muthukumar , and P. V. Ravindra . 2021. “Preeclampsia: Pathophysiology and Management.” Journal of Gynecology Obstetrics and Human Reproduction 50, no. 2: 101975. 33171282 10.1016/j.jogoh.2020.101975

[cre270177-bib-0015] Phipps, E. , D. Prasanna , W. Brima , and B. Jim . 2016. “Preeclampsia: Updates in Pathogenesis, Definitions, and Guidelines.” Clinical Journal of the American Society of Nephrology 11, no. 6: 1102–1113. 27094609 10.2215/CJN.12081115 PMC4891761

[cre270177-bib-0016] Rahmani, R. , S. Ahrari , A. Mahmoudian , N. Dadpour , M. Taghany , and M. Beshagh . 2013. “The Relationship Between Pre‐Eclampsia and Periodontal Infection in Pregnant Women.” Journal of Research and Health 3, no. 4: 514–518.

[cre270177-bib-0017] Soucy‐Giguère, L. , A. Tétu , S. Gauthier , et al. 2016. “Periodontal Disease and Adverse Pregnancy Outcomes: A Prospective Study in a Low‐Risk Population.” Journal of Obstetrics and Gynaecology Canada 38, no. 4: 346–350. 27208603 10.1016/j.jogc.2016.02.012

[cre270177-bib-0018] Tiznobaik, A. , S. Taheri , P. Torkzaban , et al. 2019. “Relationship Between Dental Plaque Formation and Salivary Cortisol Level in Pregnant Women.” European Oral Research 53, no. 2: 62–66. 31309195 10.26650/eor.20192484 PMC6614692

[cre270177-bib-0019] Wheeler, S. M. , S. O. Myers , G. K. Swamy , and E. R. Myers . 2022. “Estimated Prevalence of Risk Factors for Preeclampsia Among Individuals Giving Birth in the US in 2019.” JAMA Network Open 5, no. 1: e2142343‐e. 34982156 10.1001/jamanetworkopen.2021.42343 PMC8728614

[cre270177-bib-0020] World Health Organization (WHO) . 2013. Oral Health Surveys: Basic Methods. World Health Organization.

